# Validation of blood pressure measurement using a smartwatch in patients with acute ischemic stroke

**DOI:** 10.1097/MD.0000000000042899

**Published:** 2025-08-15

**Authors:** Yu-Hsuan Liu, Li-Ching Chen, Yi-Shian Guo, Huan-Jan Lin

**Affiliations:** a Department of Neurology, E-DA Hospital, Kaohsiung, Taiwan; b Department of Nursing, E-DA Hospital, Kaohsiung, Taiwan; c Department of Healthcare Administration, College of Medicine, I-Shou University, Kaohsiung, Taiwan; d Department of Neurology, Kaohsiung Medical University Hospital, Kaohsiung, Taiwan; e Doctoral Degree Program in Biomedical Engineering, College of Medicine, Kaohsiung Medical University, Kaohsiung, Taiwan; f Post-Baccalaureate Medicine, College of Medicine, Kaohsiung Medical University, Kaohsiung, Taiwan.

**Keywords:** acute ischemic stroke, blood pressure, smartwatch

## Abstract

We aimed to validate the accuracy of blood pressure (BP) measurement using a smartwatch in patients with acute ischemic stroke. We compared 140 pairs of BP (n = 35) measurements acquired by a smartwatch (ASUS VivoWatch SP) with those measured by a sphygmomanometer (reference device). Differences between the smartwatch BP and reference BP measurements were compared. The validation procedure and criterion followed the consensus of the American National Standards Institute, Inc/Association for the Advancement of Medical Instrumentation/International Organization for Standardization (ANSI/AAMI/ISO) 81060-2:2018 and extended the standard to the specificities of cuffless devices in acute ischemic stroke population. The mean and standard deviation of the differences measured by a smartwatch and reference device were 1.8 ± 5.7 mm Hg in systolic BP and 0.7 ± 3.6 mm Hg in diastolic BP according to criterion 1. The mean and standard deviation of the differences measured by a smartwatch and reference device were 1.8 ± 5.6 mm Hg in systolic BP and 0.7 ± 3.6 mm Hg in diastolic BP according to criterion 2. The results both met the standards of the 2 criterion. The validation result did not differ between the paralytic and non-paralytic arms. The smartwatch with photoplethysmography sensors can provide accurate and reliable measurement of BP in acute ischemic stroke patients.

## 1. Introduction

Regular and continuous blood pressure (BP) monitoring is crucial in the acute stage of ischemic stroke. The BP measured within 3 days is associated with diseased survival and functional outcome.^[1]^ Although the ideal goal of blood pressure control is still under debate in the acute stage of ischemic stroke,^[2]^ ambulatory blood pressure monitoring may provide more information for physicians in the adjustment of anti-hypertensive medication. Traditional BP measurement was typically performed by nurses from 4 to 6 times per day in the ward, and most of them were performed when patients were awake. These scarce measurements may not detect BP fluctuation across the day and are unable to obtain the BPs during sleep. These drawbacks of cuff-based BP measurement hampered the physician from documenting the physiological BP variation in 1 day. Lacking the understanding of the physiological variability of BP makes it difficult to establish a guide for BP control in the acute stage of ischemic stroke.

Recently, cuffless, wristwatch-type devices that can measure BP using photoplethysmography (PPG) sensors have been introduced in clinical practice. PPG can capture the pulse wave figuration and calculate pulse wave velocity, which can be transferred to BP values by a multivariate regression model with a trained machine learning algorithm.^[3]^ With the improvement of facility design, some devices use double PPG sensors,^[4]^ or in combination with an electrocardiography (ECG) signal,^[5]^ which can provide more accurate estimating of BP. Smartwatches, which incorporate the function of physiological data measurement and digital storage give physicians and patients opportunities to monitor BP more conveniently. In other words, the measurement of the BP with a smartwatch facilitates BP monitoring in patients anytime and anywhere.

In the pursuit of applying smartwatches for BP measurement, validation studies involving smartwatches from various manufacturers alongside a conventional sphygmomanometer were conducted.^[6–8]^ These studies yielded predominantly acceptable and accurate results. It is noteworthy that most of these validation studies were carried out in the general population, which facilitated participant recruitment. However, the validation of extremely high or low BP values, occurrences more likely in individuals with specific health conditions, was lacking, despite the heightened importance of such data. In instances where patients experienced severe hypertension or hypotension, it was crucial for the smartwatch to accurately capture the BP value and promptly provide feedback.

Patients suffering from acute ischemic stroke often exhibit fluctuating BP levels upon admission.^[9]^ This inherent variability in BP makes them a fitting cohort for assessing the smartwatch’s capability in detecting extreme blood pressure values. Therefore, in the present study, we validated the accuracy and reliability of BP measurement using a smartwatch in patients with acute ischemic stroke.

## 2. Methods

### 2.1. Participants

Patients with acute ischemic stroke were recruited from the ward of Neurology, E-DA hospital, from April 2022 to July 2022. The diagnosis of ischemic stroke was made by a board-certified neurologist, with further brain magnetic resonance imaging confirmed. The inclusion criteria were adults with age > 18 years and had an acute ischemic stroke within 3 days of onset. The exclusion criteria were as follows: patients with known aortic dissection or peripheral arterial occlusive disease history, who experienced consciousness disturbance, had a hemodynamically unstable condition, had dyspnea with oxygen use, severe immobility or other conditions that make them not able to cooperate with the BP measurement of the smartwatch.

We collected demographic and medical information of the participants, including age, sex, body mass index (BMI), wrist circumferences, and documented stroke risk factors including hypertension, diabetes mellitus, dyslipidemia, atrial fibrillation, and smoking status. This study conformed to the guidelines of the Declaration of Helsinki and received approval from the Institutional Review Board of E-DA Hospital (EMRP110096). Upon admission, written informed consent was obtained from either patients or their immediate family, and they were notified that the consent could be withdrawn without any reason afterward.

### 2.2. Test device

**Figure 1.**
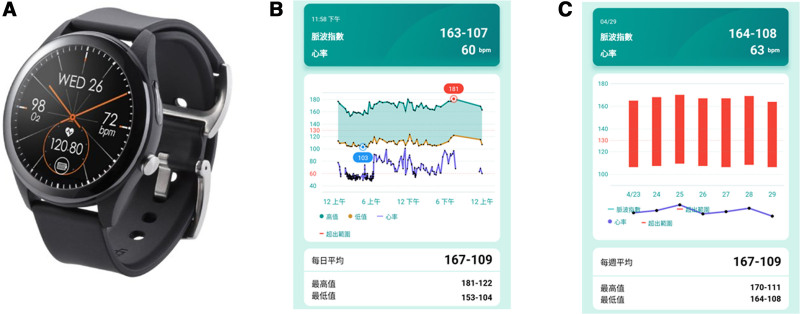
Appearance of the ASUS VivoWatch SP (A), daily BP (B), and weekly BP (C) in the ASUS HealthConnect App. BP = blood pressure.

ASUS VivoWatch SP (ASUS, Taipei, Taiwan), a smartwatch released on August 4, 2020, which was used for BP and ECG recording, was approved as a medical device by the Taiwan Ministry of Food and Drug Safety. The device also measured blood oxygen saturation, and sleep time with different stages, and steps. The device consisted of 2 PPG sensors located on the back and left side of the watch and 1 ECG sensor on the right side. The PPG sensors use an optical chip (TI AFE4900) for signal acquisition and electronic recording. The device recorded green light reflection PPG signals at 250 Hz for the current investigation. The captured pulse wave morphology and velocity were transformed to blood pressure by a multivariate logistic regression model and trained by the machine learning algorithm. The recording of BP with the smartwatch should be calibrated before measurement according to the manufacturer’s user manual. Spontaneous measurement of BP can be set at 5 minutes to 120 minutes. The smartwatch was connected to a smartphone with Bluetooth low energy 4.0. The recorded physiological data was stored within the smartphone through ASUS HealthConnect App, with different language versions. Figure 1A–C illustrates the device and sample of the recorded data in the App.

### 2.3. BP measurement protocol

**Figure 2.**
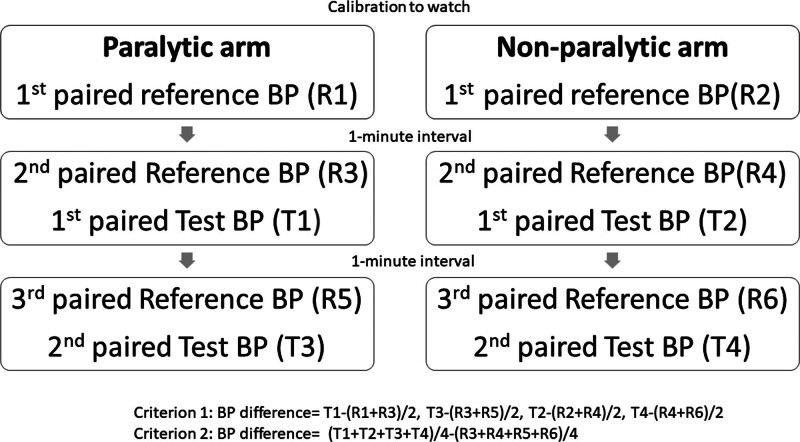
Blood pressure measurement protocol for validation. BP = blood pressure.

The devices were validated according to the sequential method of the consensus.^[10]^ The BP measurements were performed in the acute stage of admission (within 2 days of admission before any anti-hypertensive agents use). Patients were brought to a quiet room and started the validation procedure after a 5-minute rest. Two smartwatches were equipped 2 cm away from both wrists of the patient independently and linked to the connected smartphone. The wrists of the patients were placed at the level of the heart to prevent confounding effects related to hydrostatic pressure differences. The observers fit the BP measuring cuffs on both the paralytic and non-paralytic arms. Three cuff sizes were chosen according to the mid-arm circumference: a small cuff for arm circumferences of 18 to 23 cm; a standard cuff of 24 to 32 cm; and a large cuff of 32 to 42 cm. Two board-certified nurses simultaneously measured the first reference BPs of both arms with a standard mercury sphygmomanometer. The reference systolic blood pressure (SBP) was determined based on the phase I Korotkoff sound and diastolic blood pressure (DBP) was determined based on the phase V Korotkoff sound. The first paired reference BP data was entered into the ASUS HealthConnect App for calibration, as requested by the manufacturer. Then, the nurses again obtained the second paired reference BP with an interval of 1 minute at both arms, and the first paired test BP was obtained from the smartwatch simultaneously. The third paired reference BP and the second paired test BP were obtained after another 1-minute interval. The measured BPs were labeled independently as a reference and test BP of paralytic limbs and non-paralytic arms. The BP measurement protocol is presented in Figure 2.

### 2.4. Statistical analyses

Data were analyzed according to criterion 1 and 2 of the validation consensus.^[10]^ For criterion 1, we calculated the BP differences defined as the SBP or DBP value of the automatic test device minus the mean value of the SBP or DBP measured by the mercury sphygmomanometer before and after the BP measurement by the test device. Four tests and 6 reference BP values were determined for each participant. The mean value and standard deviation (SD) of these difference values were calculated. The mean error of all valid paired BP should be ≤ 5 mm Hg with an SD < 8 mm Hg. For criterion 2, we calculated the BP differences defined as the mean value of 4 SBPs or DBPs measured by the test device minus the mean value of 6 reference SBPs or DBPs. The mean and SD of these difference values were calculated. The SDs of SBP should be < 6.95 mm Hg and DBP should be < 6.82 mm Hg.

According to the consensus, the acquired reference SBP with the percentages of high (≥160 mm Hg), medium (≥140 mm Hg), and low (≤100 mm Hg) must exceed 5%, 20%, and 5%, respectively. For reference DBP, the percentages of high (≥100 mm Hg), medium (≥85 mm Hg), and low DBP (≤60 mm Hg) must exceed 5%, 20%, and 5%, respectively. Bland–Altman plots were used to show deviations in the data. Statistical analyses were performed using MedCalc statistical software version 20.111 (MedCalc Software Ltd, Ostend, Belgium).

### 2.5. Adjustment of the validation protocol

In alignment with the protocol outlined in ISO81060-2:2018, some adjustments were implemented. To elucidate these differences, we have provided a comparative overview of our protocol and the consensus protocol in Table 1. Firstly, we opted for a sample size of N = 35, tailored specifically to the unique population of acute ischemic stroke patients, as opposed to the N = 85 recommended for the general population. Secondly, we refrained from assigning cuff size-stratified subgroups, recognizing that this parameter was not applicable to a smartwatch. Lastly, a minor modification was made to the validation procedure for BP measurement. Instead of employing a one-arm sequential measurement, we conducted simultaneous two-arm sequential measurements. This allowed us to assess the accuracy of the smartwatch in both paralytic and non-paralytic arms. It is essential to note that the consensus document stipulated that the standard protocol was designed exclusively for cuff-based BP measurement. Separate validation protocols will be devised for continuous, cuffless, and central BP monitors, as outlined in the section titled “Validation of other BP Monitors.”^[10]^

**Table 1:** Comparison of the consensus and the study protocol in BP measurement.

	Consensus protocol	Study protocol
Validation sample size	At least 85 subjects. In case an independent general population of 85 subject studies has been completed successfully, a compromise was agreed to accept a minimum of 35 special population subjects.	35 subjects in the acute ischemic stroke population.
Cuff size-stratified subgroups	A minimum of 22 subjects per cuff, which means, that 4 cuffs could be evaluated in an 88-subject study.	Not applicable in the watch device.
Reference blood pressure measurement and validation procedure	Same arm sequential measurement. Four reference BP measurements. follow, alternated by 3 test device measurements (R1–T1–R2–T2–R3–T3–R4). Measurements will be performed with at least 60-second intervals.	Same arm sequential measurement with 2 arms performed simultaneously. Divided into 2 arms, first reference BP measurements and device calibration, followed by 2 reference BP measured simultaneously with 2 test devices. Measurement performed with 60-second intervals.
Validation criteria	BP difference meets both criteria 1 and 2. Presented with Bland–Altman scatterplots.	BP difference meets both criteria 1 and 2. Presented with Bland–Altman scatterplots.

## 3. Results

A total of 38 acute ischemic stroke patients received the validation procedure. Three patients were excluded because they failed to obtain blood pressure from the smartwatch, and the situation was unable to be corrected after increased skin moisture and adjusting the tightness of the band of the smartwatch. Therefore, 35 patients were enrolled to conduct the final analysis. The demographic characteristics and the result of reference BP measurement of the 35 patients are shown in Table 2. The percentage of the high, medium, and low reference BPs fulfilled the criteria of the consensus.

**Table 2:** Clinical characteristics and demographics of the study population.

	Values
Number of patients included	35
Number of BP paired	140
Age	67.4 ± 9.7
Male (%)	25 (71.4%)
BMI	25.8 ± 3.2
Wrist circumferences (cm)	18.5 ± 0.8
Hypertension (%)	27 (77.1%)
Diabetes mellitus (%)	20 (57.1%)
Dyslipidemia (%)	28 (80.0%)
Atrial fibrillation (%)	4 (11.4%)
Smoking (%)	13 (37.1%)
SBP	
≥160 mm Hg (%)	18 (12.9%)
≥140 mm Hg (%)	37 (26.4%)
≤100 mm Hg (%)	11 (7.9%)
DBP	
≥100 mm Hg (%)	29 (20.7%)
≥85 mm Hg (%)	68 (48.6%)
≤60 mm Hg (%)	9 (6.4%)
Heart rate	75.2 ± 13.5

Data are expressed as the number (%) or mean ± standard deviation.

BMI = body mass index, DBP = diastolic blood pressure, SBP = systolic blood pressure.

The mean SBP and DBP measured by the smartwatch were 132.6 mm Hg (range: 96–211 mm Hg) and 83.9 mm Hg (range: 43–123 mm Hg), respectively. The mean heart rate measured by the smartwatch was 76.4 bpm (range: 48–112 bpm). The mean SBP and DBP of the reference device were 130.8 mm Hg (range: 89–222 mm Hg) and 83.2 (range: 44–122 mm Hg), respectively. The mean heart rate measured by the observer was 75.2 bpm (range: 48–110 bpm).

**Figure 3.**
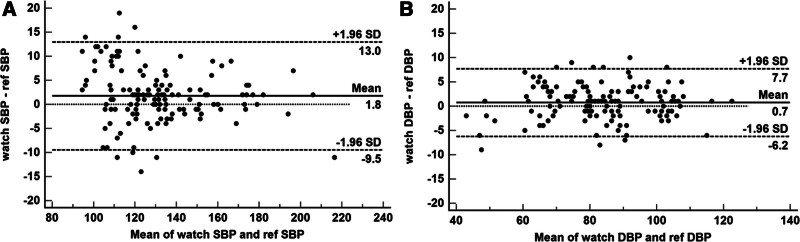
Bland–Altman plots for the differences between the smartwatch and the reference measurements for SBP (A) and DBP (B) (N = 140). DBP = diastolic blood pressure, SBP = systolic blood pressure.

The mean differences between the reference and smartwatch BP were 1.8 ± 5.7 mm Hg and 0.7 ± 3.6 mm Hg for SBP and DBP according to criterion 1. The mean differences between the reference and smartwatch BP were 1.8 ± 5.6 and 0.7 ± 3.6 mm Hg for SBP and DBP according to criterion 2. These results fulfilled the validation criteria of the consensus guideline of both criteria 1 and 2. Bland–Altman plots of SBP and DBP differences between the smartwatch and reference measurements are shown in Figure 3(A–B). The smartwatch BP and reference BP showed no significant difference between measurements, regardless of whether the smartwatch was worn on the paralytic or non-paralytic arm (Table 3).

**Table 3:** Comparison of test BP and reference BP difference between measurement in the paralytic arm and non-paralytic arm.

	Test SBP	Ref SBP	*P* value	Test DBP	Ref DBP	*P* value
Paralytic arm	132.8 ± 23.4	130.9 ± 23.8	.63	83.9 ± 16.4	83.0 ± 16.0	.75
Non-paralytic arm	132.3 ± 24.3	130.8 ± 25.8	.71	83.9 ± 15.9	83.3 ± 16.1	.82

DBP = diastolic blood pressure, Ref = reference, SBP = systolic blood pressure.

## 4. Discussion

This is the first study to validate BP measurement using the smartwatch (ASUS VivoWatch SP) in patients with acute ischemic stroke. Our study showed that using a smartwatch with PPG sensors combined with an ECG sensor is an accurate and reliable method for measuring BP in patients with acute ischemic stroke.

BP monitoring plays a critical role in the management of acute stroke, but it is essential to acknowledge that BP levels can fluctuate with different activities and over time.^[11]^ Smartwatches offer a promising avenue for monitoring BP, even during sleep without disrupting patients’ rest. It is known that BP tends to naturally decrease during various stages of sleep. Detecting non-dipping of nocturnal BP is particularly pertinent as it has significant implications for cardiovascular events.^[12,13]^ This is a physiological pattern that traditional BP measurements may not be equipped to identify, and it is worth noting that an excessive correction of BP can lead to hypotension, potentially compromising adequate brain perfusion during sleep and exacerbating neurological deficits.^[14]^ In addition to BP, smartwatches can provide a wealth of other valuable data. They leverage accelerometers to track sleep onset and distinguish between wakefulness and various sleep stages. Moreover, they can measure blood oxygen saturation, heart rate, and even perform ECGs. These physiological data can assist physicians in monitoring comorbidities like insomnia,^[15]^ obstructive sleep apnea,^[16]^ or arrhythmias^[17]^ in patients with acute ischemic stroke. This holistic approach to monitoring can provide a more comprehensive understanding of a patient’s health status, allowing for more personalized and effective care.

Wearable and cuffless devices used in special disease populations in BP measurement were scarce but emerging.^[18–20]^ A study described the influence of tremors in detecting BP with a smartwatch in Parkinson disease, and they suggest that the device wore on the less affected arm.^[21]^ Patients with acute ischemic stroke usually suffer from hemiparesis which may hamper them from wearing the watch. Moreover, the physiologic data collected from the paralytic and non-paralytic arms may show differences and they should be validated separately.^[22,23]^ Our study revealed a similar validation result of wearing the watch on paralytic arms and non-paralytic arms, and they were both accurate and reliable. The result can provide important information for future study design of smartwatches in stroke patients.

This study presents several noteworthy limitations. Firstly, our validation procedure was conducted exclusively when patients were in a state of rest and wakefulness. As such, the accuracy of the BP data obtained during physical activity and sleep remains unknown. Secondly, our exclusion criteria encompassed patients with uncooperative consciousness and those experiencing hemodynamic instability. This introduces an element of uncertainty regarding the potential influence of these conditions on the measured BP. Furthermore, the sample size employed in our study was relatively modest and did not meet the minimal requirement as outlined in the consensus.^[10]^ This was primarily due to the vulnerable nature of our participant pool, consisting of acute stroke patients. Enlisting a larger cohort of stroke volunteers proved to be a challenging endeavor. Nevertheless, we speculate that the measured BP may exhibit greater variability and higher values compared to the general population. This presumption allowed us to validate the performance of the smartwatch’s BP measurement with a limited sample size. The validation results exhibited consistent accuracy and demonstrated a broader range of measured BP, compared to those of other smartwatches tested in both general and specialized populations.^[21,24]^ Consequently, our protocol might be acceptable, particularly within the specific population of acute ischemic stroke patients.

In conclusion, BP measurement using a smartwatch with a PPG sensor is accurate and reliable for patients with acute ischemic stroke. The device might be considered a long-term monitoring tool for BP, providing informative data in clinical follow-up.

## Acknowledgments

The authors thanked all the participants of the study.

## Author contributions

**Conceptualization:** Huan-Jan Lin.

**Data curation:** Yi-Shian Guo.

**Project administration:** Yi-Shian Guo.

**Supervision:** Huan-Jan Lin.

**Validation:** Li-Ching Chen.

**Writing – original draft:** Yu-Hsuan Liu.

**Writing – review & editing:** Huan-Jan Lin.
